# Supplementary material for: A Long-Term Energy-Rich Diet Increases Prefrontal BDNF in Sprague-Dawley Rats
Source: Nutrients. 2021 Dec 28;14(1):126. doi: 10.3390/nu14010126 (PMC8746649; doi:10.3390/nu14010126)
Supplement: Supplementary file 1 [file nutrients-14-00126-s001.zip › nutrients-1511193-supplementary.pdf]

**Supplementary Table S1.** Components of the Cafeteria Diet (CAF).

| **Name of diet component** | | **Altromin 1324** | **CAF diet, summarized** | **CAF component 1: Marie biscuits** | **CAF component 2: "Drømmekage" (Dreamcake)** | **CAF component 3: "Coco Tops" (No longer available for purchase)** | **CAF component 4: "Usaltet smør" (Un-salted butter)** |
|---|---|---|---|---|---|---|---|
| Brand | | Altromin | | | Karen Volf | Karen Volf | Lurpak |
| Company/ Manufacturer | | Altromin, Germany | | Pally biscuits, The Netherlands | Bisca A/S Denmark | Bisca A/S Denmark | ARLA, Denmark |
| **Diet** | | **Control Treatment** | | **Treatment (CAF)** | **Treatment (CAF)** | **Treatment (CAF)** | **Treatment (CAF)** |
| Contents Per 100 g | | | | | | | |
| | Energy (kcal) | 318.8 | 534 | 454 | 469 | 465 | 747 |
| | Fat (g) | 4.1 | 36.3 | 13 | 25 | 25 | 82 |
| | Saturated fat (g) | 0.46 | 21.3 | 1.2 | 11 | 20 | 53 |
| | Carbohydrates (g) | 40.8 | 47.4 | 75 | 57 | 57 | 0.7 |
| | Sugar (g) | 4.9 | 27.4 | 19 | 41 | 49 | 0.7 |
| | Protein (g) | 19.2 | 3.8 | 7.7 | 2.8 | 4.1 | 0.6 |
| | Water content (g) | 10 | 16 | - | - | - | - |

The rats were fed 50 g CAF diet per cage per day for five days a week over the course of five months; The CAF diet consisted of 12.5 g of each of the four CAF components; CAF= Cafeteria Diet.
